# Supplementary material for: NUPR1 protects against hyperPARylation-dependent cell death
Source: Commun Biol. 2022 Jul 22;5:732. doi: 10.1038/s42003-022-03705-1 (PMC9307593; doi:10.1038/s42003-022-03705-1)
Supplement: Supplementary file 2 — Supplementary Information [file 42003_2022_3705_MOESM2_ESM.pdf]

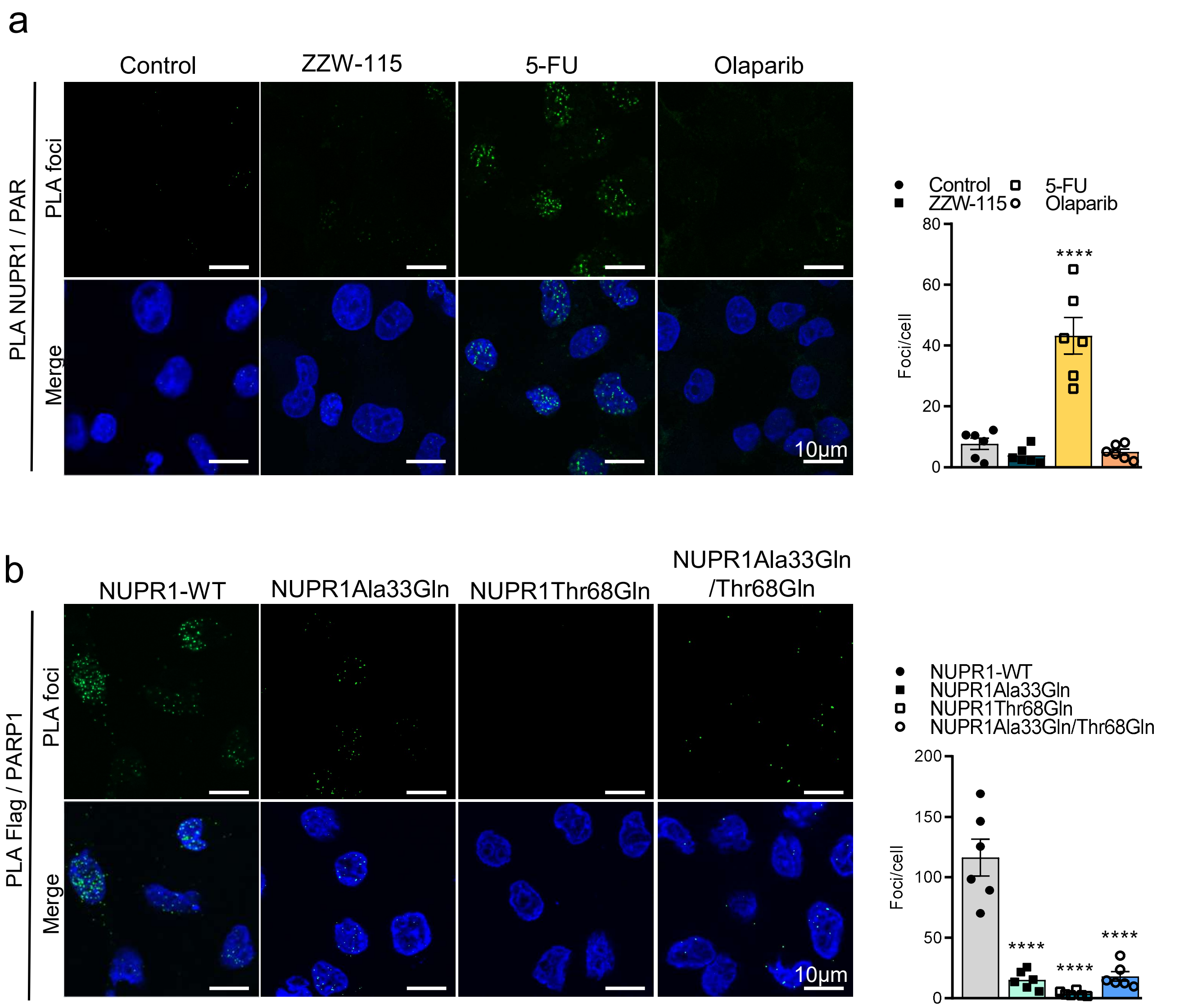

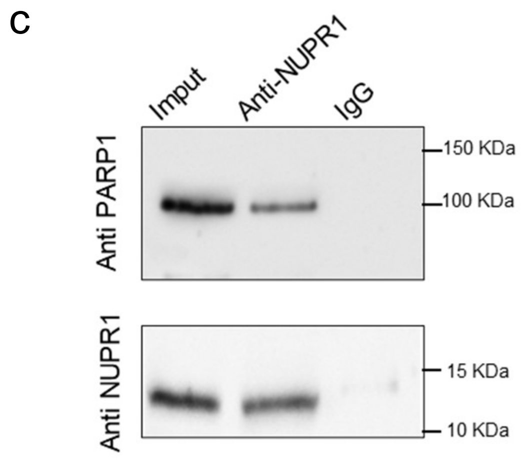

Supplementary Figure 1. PLA was performed in MiaPaCa-2 cells in the presence or in the absence of ZZW-115 at 1.5 µM, 5-FU at 10 µM, olaparib at 25µM 24 hours. Mouse anti-PAR and rabbit anti-NUPR1 antibodies were used. PLA was performed in MiaPaCa-2 cells transfected with NUPR1-Flag constructs (WT, Ala33Gln, NUPR1Thr68Gln and NUPR1 Ala33Gln/Thr68Gln). Mouse anti-Flag and rabbit anti-PARP1 antibodies were used. A representative experiment is shown (n = 6). ImageJ was used to count the number of green dots. Data represent mean ± SEM. One-way ANOVA, Dunnett correction; *P < 0.05, **P < 0.01, ***P < 0.001, ****P < 0.001. (C) PARP1/NUPR1direct interaction by co-IP was evaluated by pulling down with an anti-NUPR1 antibody and immunoblotted with anti-PARP1 (top) or anti-NUPR1 (bottom). IgG control was used.

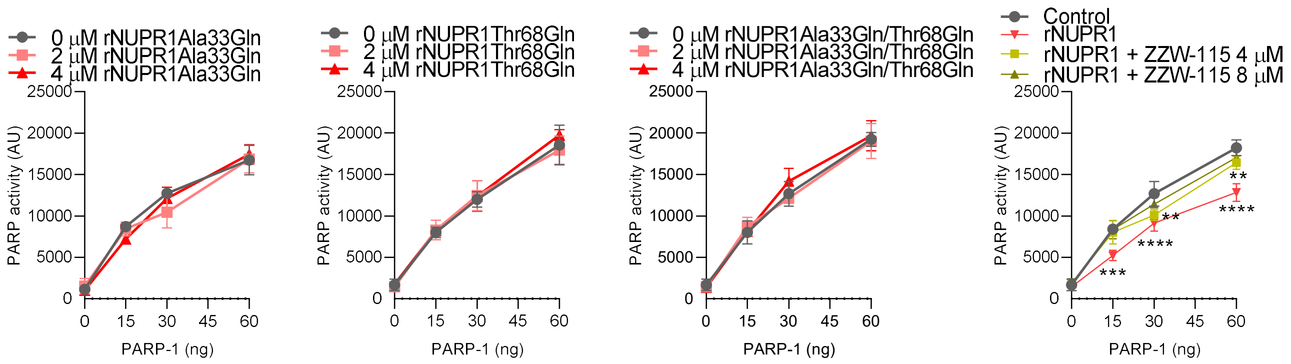

Supplementary Figure 2

PARP1 enzymatic activity was measured *in vitro* alone or in combination with 2 or 4 µM of mutants recombinant NUPR1 proteins (Thr68Gln, Ala33Gln, Thr68Gln/Ala33Gln) or recombinant NUPR1-WT in the presence of ZZW-115 (n = 3). Data represent mean ± SEM. Two-way ANOVA with Sidak correction *$P < 0.05$, **$P < 0.01$, ***$P < 0.001$, ****$P < 0.001$.

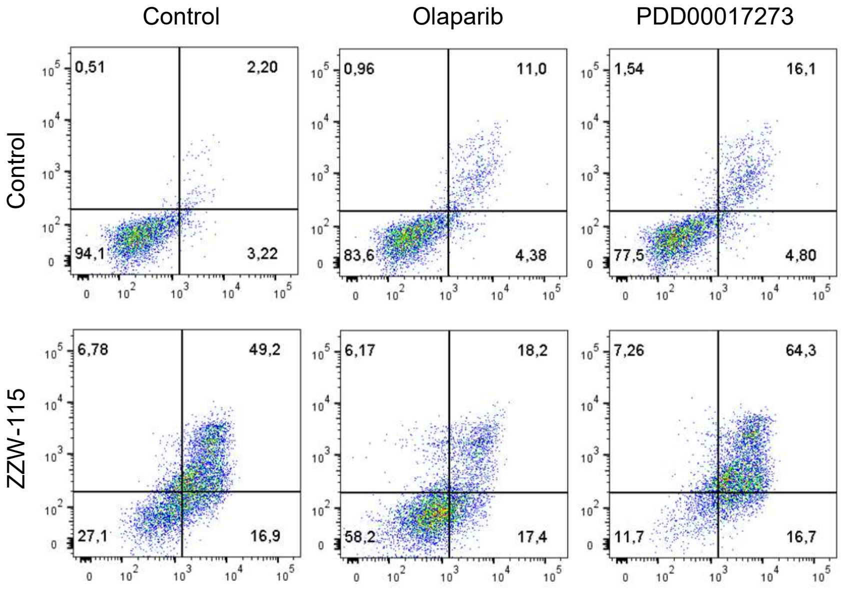

Supplementary Figure 3

NUPR1 inhibition-induced cell death was rescued by PARP1 inhibitor and enhanced by PARG inhibitor. Flow cytometry analysis of annexin V/PI staining following 24 hours of treatment with 3 µM ZZW-115 in the presence or absence of olaparib (25 µM) or PDD00017273 (1 µM). A representative experiment of the dot plot profile of cells is shown (n = 3).

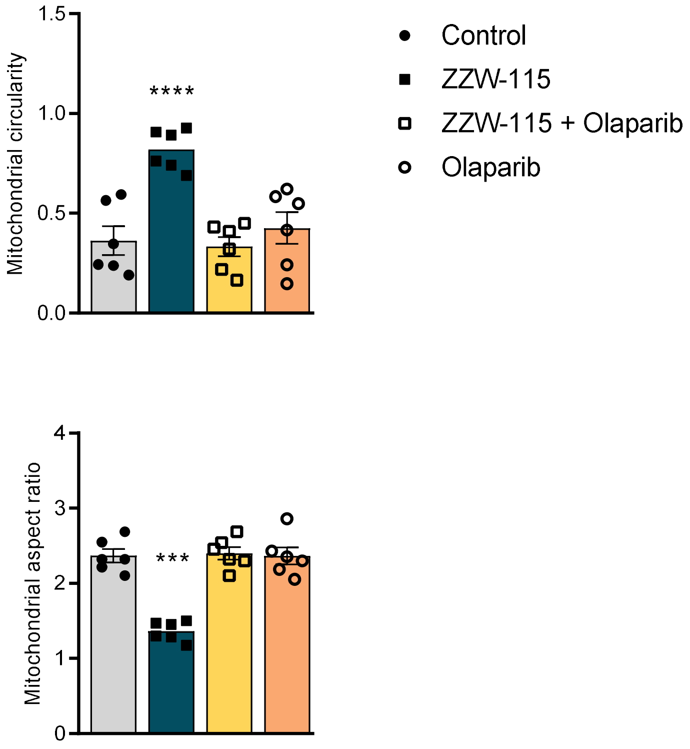

Supplementary Figure 4

ZZW-115-induced mitochondrial dysfunction was reversed by PARP1 inhibitors. Mitochondrial circularity and mitochondrial aspect ratio were calculated in MiaPaCa-2 cells were treated with ZZW-115 (1.5 µM) and olaparib (25 µM) or both for 24 h, then, loaded with MitoTracker Deep Red FM. ImageJ 1.53C (NIH) was used for analysis. Data represent mean ± SEM. One-way ANOVA, Dunnett correction; *$P < 0.05$, **$P < 0.01$, ***$P < 0.001$, ****$P < 0.001$ (n=6).

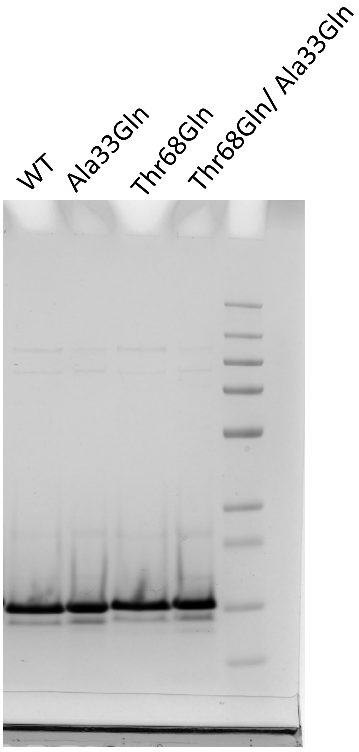

Supplementary Figure 5. Protein Expression and Purification of WT and mutants NUPR1 proteins. A denaturing SDS-PAGE/coommasie was used.

Figure 1B

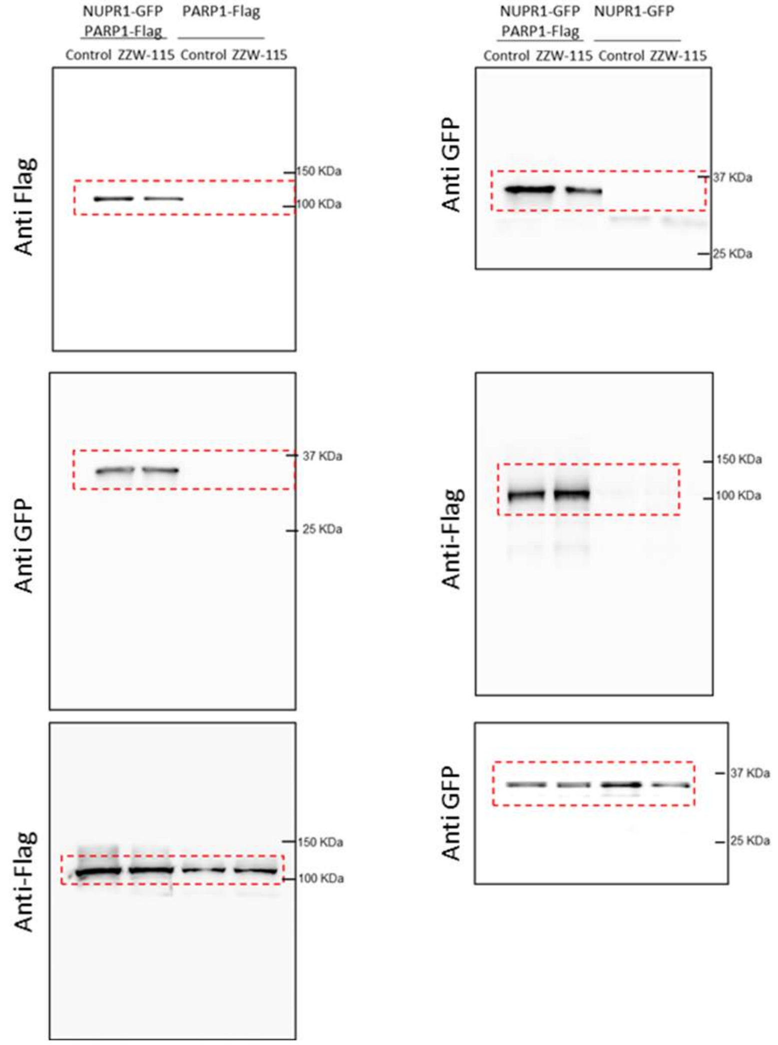

Figure 2D

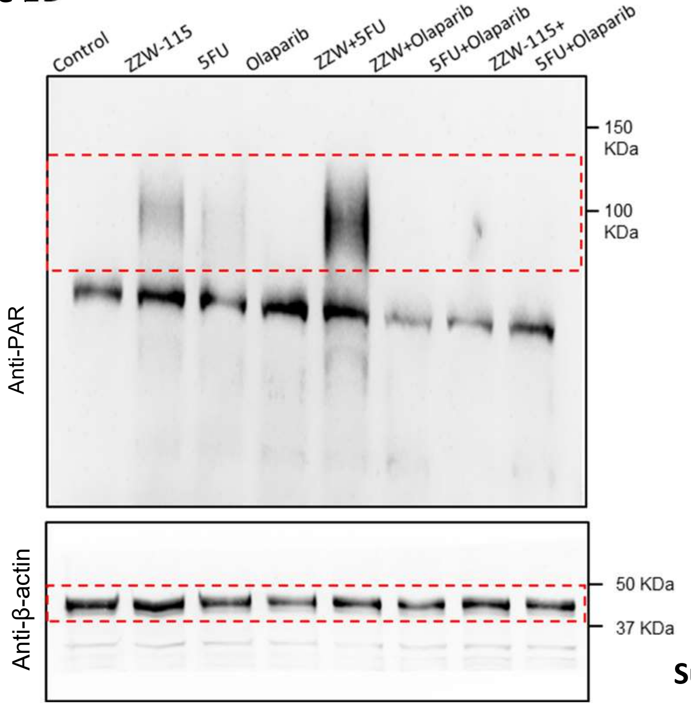

Sup. Figure 1A

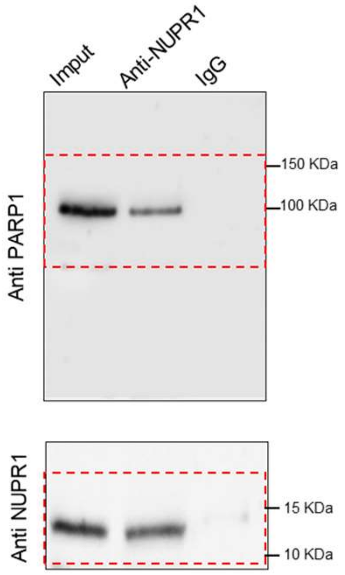

Supplementary Figure 6. Uncropped blots
