# Supplementary material for: NUPR1 protects against hyperPARylation-dependent cell death
Source: Commun Biol. 2022 Jul 22;5:732. doi: 10.1038/s42003-022-03705-1 (PMC9307593; doi:10.1038/s42003-022-03705-1)
Supplement: Supplementary file 3 — Description of Additional Supplementary Files [file 42003_2022_3705_MOESM3_ESM.pdf]

Description of Additional Supplementary Files

File name: Supplementary Data 1
Description: NUPR1-associated proteins were identified by mass spectrometry resulting in 656 Flag-tagged NUPR1-interacting proteins under normal conditions, and 1152 proteins under glucose starvation.

File name: Supplementary Data 2
Description: NUPR1-associated proteins were identified by mass spectrometry resulting in 271 and 530 proteins for GFP-tagged NUPR1 under normal growth conditions and glucose starvation, respectively.
