# Supplementary material for: NUPR1 protects against hyperPARylation-dependent cell death
Source: Commun Biol. 2022 Jul 22;5:732. doi: 10.1038/s42003-022-03705-1 (PMC9307593; doi:10.1038/s42003-022-03705-1)
Supplement: Supplementary file 6 — Reporting Summary [file 42003_2022_3705_MOESM6_ESM.pdf]

nature portfolio

Corresponding author(s): Juan Iovanna

Last updated by author(s): May 10, 2022

Reporting Summary

Nature Portfolio wishes to improve the reproducibility of the work that we publish. This form provides structure for consistency and transparency in reporting. For further information on Nature Portfolio policies, see our Editorial Policies and the Editorial Policy Checklist.

Statistics

For all statistical analyses, confirm that the following items are present in the figure legend, table legend, main text, or Methods section.

n/a	Confirmed	
☐	☒	The exact sample size (*n*) for each experimental group/condition, given as a discrete number and unit of measurement
☐	☒	A statement on whether measurements were taken from distinct samples or whether the same sample was measured repeatedly
☐	☒	The statistical test(s) used AND whether they are one- or two-sided<br>*Only common tests should be described solely by name; describe more complex techniques in the Methods section.*
☐	☒	A description of all covariates tested
☐	☒	A description of any assumptions or corrections, such as tests of normality and adjustment for multiple comparisons
☐	☒	A full description of the statistical parameters including central tendency (e.g. means) or other basic estimates (e.g. regression coefficient) AND variation (e.g. standard deviation) or associated estimates of uncertainty (e.g. confidence intervals)
☒	☐	For null hypothesis testing, the test statistic (e.g. *F*, *t*, *r*) with confidence intervals, effect sizes, degrees of freedom and *P* value noted<br>*Give P values as exact values whenever suitable.*
☒	☐	For Bayesian analysis, information on the choice of priors and Markov chain Monte Carlo settings
☒	☐	For hierarchical and complex designs, identification of the appropriate level for tests and full reporting of outcomes
☒	☐	Estimates of effect sizes (e.g. Cohen's *d*, Pearson's *r*), indicating how they were calculated

Our web collection on statistics for biologists contains articles on many of the points above.

Software and code

Policy information about availability of computer code

Data collection	*Provide a description of all commercial, open source and custom code used to collect the data in this study, specifying the version used OR state that no software was used.*
Data analysis	*Provide a description of all commercial, open source and custom code used to analyse the data in this study, specifying the version used OR state that no software was used.*

For manuscripts utilizing custom algorithms or software that are central to the research but not yet described in published literature, software must be made available to editors and reviewers. We strongly encourage code deposition in a community repository (e.g. GitHub). See the Nature Portfolio guidelines for submitting code & software for further information.

Data

Policy information about availability of data

All manuscripts must include a data availability statement. This statement should provide the following information, where applicable:
- Accession codes, unique identifiers, or web links for publicly available datasets
- A description of any restrictions on data availability
- For clinical datasets or third party data, please ensure that the statement adheres to our policy

Provide your data availability statement here.

Field-specific reporting

Please select the one below that is the best fit for your research. If you are not sure, read the appropriate sections before making your selection.

☒ Life sciences ☐ Behavioural & social sciences ☐ Ecological, evolutionary & environmental sciences

For a reference copy of the document with all sections, see nature.com/documents/nr-reporting-summary-flat.pdf

Life sciences study design

All studies must disclose on these points even when the disclosure is negative.

Sample size	*Describe how sample size was determined, detailing any statistical methods used to predetermine sample size OR if no sample-size calculation was performed, describe how sample sizes were chosen and provide a rationale for why these sample sizes are sufficient.*
Data exclusions	*Describe any data exclusions. If no data were excluded from the analyses, state so OR if data were excluded, describe the exclusions and the rationale behind them, indicating whether exclusion criteria were pre-established.*
Replication	*Describe the measures taken to verify the reproducibility of the experimental findings. If all attempts at replication were successful, confirm this OR if there are any findings that were not replicated or cannot be reproduced, note this and describe why.*
Randomization	*Describe how samples/organisms/participants were allocated into experimental groups. If allocation was not random, describe how covariates were controlled OR if this is not relevant to your study, explain why.*
Blinding	*Describe whether the investigators were blinded to group allocation during data collection and/or analysis. If blinding was not possible, describe why OR explain why blinding was not relevant to your study.*

Reporting for specific materials, systems and methods

We require information from authors about some types of materials, experimental systems and methods used in many studies. Here, indicate whether each material, system or method listed is relevant to your study. If you are not sure if a list item applies to your research, read the appropriate section before selecting a response.

Materials & experimental systems

n/a	Involved in the study	
☐	☒	Antibodies
☐	☒	Eukaryotic cell lines
☒	☐	Palaeontology and archaeology
☒	☐	Animals and other organisms
☒	☐	Human research participants
☒	☐	Clinical data
☒	☐	Dual use research of concern

Methods

n/a	Involved in the study	
☒	☐	ChIP-seq
☐	☒	Flow cytometry
☒	☐	MRI-based neuroimaging

Antibodies

Antibodies used	anti-GFP from rabbit, from Invitrogen, ref A-11122; anti-Flag from mouse, MilliporeSigma, ref F1804; anti-NUPR1 antibody from rabbit, homemade; anti-PAR antibody from mouse, Abcam, ref ab14459; anti-Mitofusin2 from Cell Signaling, ref 9482; anti-AIF antibody from Cell Signaling, ref 4642; anti-Poly (ADP-Ribose) Polymer antibody from mouse, Abcam, ref ab14459; anti-β-actin from mouse, Sigma, ref A5316; secondary antibodies : goat anti-mouse Alexa Fluor 488, ref A28175, and goat anti-rabbit Alexa Fluor 488, ref A27034, both from Thermo Fisher Scientific.
Validation	western blotting, IF and / or IHC

Eukaryotic cell lines

Policy information about cell lines

Cell line source(s)	MiaPaCa-2 cells were obtained from American Type Culture Collection (ATCC, USA)
Authentication	not autentified
Mycoplasma contamination	cells were free of mycoplasma

Commonly misidentified lines
(See ICLAC register)

Name any commonly misidentified cell lines used in the study and provide a rationale for their use.

Flow Cytometry

Plots

Confirm that:

☒ The axis labels state the marker and fluorochrome used (e.g. CD4-FITC).

☒ The axis scales are clearly visible. Include numbers along axes only for bottom left plot of group (a 'group' is an analysis of identical markers).

☒ All plots are contour plots with outliers or pseudocolor plots.

☒ A numerical value for number of cells or percentage (with statistics) is provided.

Methodology

Sample preparation

Measurement of ROS and mitochondrial ROS: Cells were seeded at 8 x 104 cells per well in 24-well plates. Next day, cells were treated with olaparib and indicated concentrations of ZZW-115 for 24 h. After that, cells were incubated with 5 μM CellROX Green Reagent (C10444, Thermo, USA) or 10 μM MitoSOX Red (M36008, Thermo, USA) at 37°C for 30 minutes in the dark. Then, the unincorporated dye was removed by washings with prewarmed PBS.
Mitochondrial membrane potential assay : Mitochondrial membrane potential assay was performed using MitoProbe TMRM Assay Kit (M20036, Invitrogen) following the manufacturer's protocol. After 24 hours of incubation in presence of ZZW-115 and or olaparib, cells were dissociated using accutase (Thermo Fisher) and resuspended in 200μL PBS at the density of 1x106 cells/mL. Then, we added 1μL of 20μM stock TMRM reagent solution to the cells and incubate for 30minutes at 37°C, 5% CO2.
Cytosolic calcium concentration : Cells were seeded at 8 x 104 cells per well in 24-well plates. After 24 hours of treatment, cells were preincubated with Fluo-4 AM (Molecular Probes) for 30minutes at 37°C. Then, cells were washed and resuspended in HBSS for flow cytometry analysis.
AnnexinV/PI staining : Cells were collected after incubation for 24 hours of treatment. Cells were washed and then detached with Accutase, and resuspended in annexin-binding buffer. Pacific-Blue annexin V (5 μl, BioLegend) was added to the cell suspension and incubated for 15 minutes. Before analysis by flow cytometry, propidium iodide (5 μl, Miltenyi Biotec) was added to the suspension

Instrument

MACSQuant-VYB (Miltenyi Biotec, Surrey, UK)

Software

FlowJo 10.7.1 software

Cell population abundance

Ten thousand events per sample were collected

Gating strategy

Describe the gating strategy used for all relevant experiments, specifying the preliminary FSC/SSC gates of the starting cell population, indicating where boundaries between "positive" and "negative" staining cell populations are defined.

☒ Tick this box to confirm that a figure exemplifying the gating strategy is provided in the Supplementary Information.
